# Quantitative Evaluation of Twelve Major Components of Sulfur-Fumigated Astragali Radix with Different Durations by UPLC-MS

**DOI:** 10.3390/molecules23102609

**Published:** 2018-10-11

**Authors:** Xiaoyan Xing, Zhonghao Sun, Meihua Yang, Nailiang Zhu, Junshan Yang, Guoxu Ma, Xudong Xu

**Affiliations:** Key Laboratory of Bioactive Substances and Resource Utilization of Chinese Herbal Medicine, Ministry of Education, Beijing Key Laboratory of Innovative Drug Discovery of Traditional Chinese Medicine (Natural Medicine) and Translational Medicine, Key Laboratory of Efficacy Evaluation of Chinese Medicine against Glycolipid Metabolic Disorders, State Administration of Traditional Chinese Medicine, Institute of Medicinal Plant Development, Peking Union Medical College and Chinese Academy of Medical Sciences, Beijing 100193, China; xyxing@implad.ac.cn (X.X.); sun_zhonghao@126.com (Z.S.); mhyang@implad.ac.cn (M.Y.); nlzhu@implad.ac.cn (N.Z.); jsyang@implad.ac.cn (J.Y.)

**Keywords:** Astragali Radix, sulfur-fumigation duration, quantification, synthesis

## Abstract

In this study, an improved UPLC-MS (Ultra-high performance liquid chromatography-tandem mass spectrometry) method for simultaneously quantifying twelve major components belonging to two chemical types was developed and validated, and was applied to quantitatively compare the quality of sulfur-fumigated Astragali Radix of different durations and of the fresh reference sample. The results showed that the contents of triterpenes astragaloside III and astragaloside IV decreased moderately, while the flavonoids calycosin, formononetin, and 7,2′-dihydroxy-3′,4′-dimethoxyisoflavane decreased significantly. The corresponding flavonoid glycosides increased accordingly, which indicated the occurrence of chemical transformation of flavonoids and glycosides in the process of sulfur-fumigation. These transformations were further confirmed by the synthesis of flavonoid glycosides under simulated sulfur-fumigation circumstances. Furthermore, the sulfur-fumigated duration varied in proportion with the contents of compounds **7**, **11**, and **12**. These results suggest that the established method was precise, accurate and sensitive enough for the global quality evaluation of sulfur-fumigated Astragali Radix. Further, sulfur-fumigation not only changes the proportions of bioactive components, but also causes chemical transformation in Astragali Radix.

## 1. Introduction

Astragali Radix (AR), the dry roots of *Astragalus membranaceus* (Fisch.) Bunge. or *Astragalus mongholicus* Bunge., is one of the most widely used “Qi-tonifying” Chinese herbal medicines. In the doctrines of traditional Chinese medicines (TCM), AR is claimed to have beneficial effects, including “tonifying the Qi” of the kidney [1], arresting sweating, “tonifying Qi” and “lifting yang”, inducing diuresis to alleviate edema, relieving fever with “sweet and warm-natured” drugs, promoting wound healing and tissue regeneration [2]. Combined with pharmacological studies, AR has been used in clinics to treat diabetic and reduce the risk of diabetic complications [3], cardio-cerebrovascular disease, respiratory disease, and digestive system disease [4], due to its immunomodulation [5], anti-inflammation [6], anti-tumor [7], nerve cell protecting and recovery [8], anti-aging, and cardioprotective effects [9]. Previous research found that the main active ingredients of AR include flavonoids and isoflavones, saponins, polysaccharides, and others [10]. Traditionally, the post-harvest processing of the roots of AR consists of sun-drying the whole fresh root after cleaning. Because it is prone to mildew attack, AR has been recently reported as being sulfur-fumigated during post-harvesting handling prior to storage. So as a consequence it has become necessary to compare the variations in AR chemical profiles after the sulfur-fumigation. Sulfur-fumigation, which is an easy to apply and low-cost operation, has been commonly used to prevent medicinal herbs from pest infestation, mold, and bacterial contamination [11]. However, recent studies demonstrated that this method could leave residue of hazardous substances such as sulfur dioxide and heavy metals, which posed a threat to human health [12]. Furthermore, sulfur-fumigation was reported to reduce the content of the active ingredients in herbs, and influence the chemical transformation of bioactive components and even to alleviate the pharmacological activities of edible herbs [13,14,15]. In 2004, the State Food and Drug Administration of China indicated that sulfur fumigated medicinal herbs are inferior [16]. However, the sulfur fumigation treatment of medicinal herbs and foods still prevails all over the world, which exerts a negative impact on the safe application of edible herbs. To the best of our knowledge, few systemic studies have been reported on the quantitative evaluation of sulfur-fumigated AR. In particular, the durational effects of sulfur-fumigation on the proportions of bioactive components in AR have not been quantitatively evaluated. 

**Figure 1:**
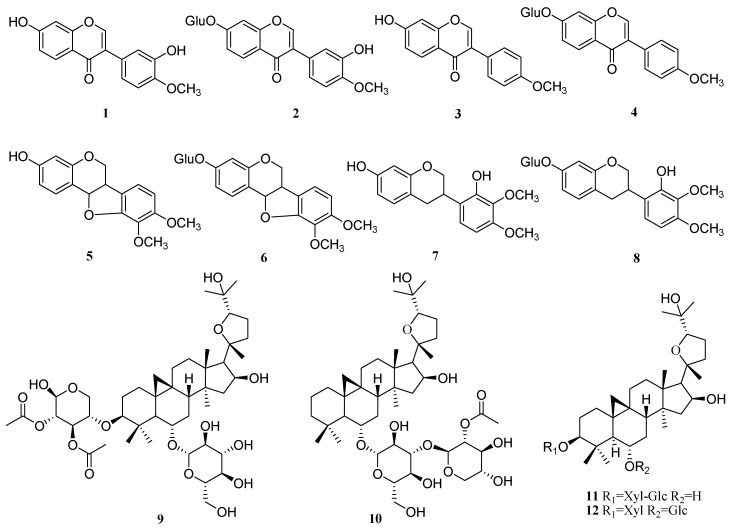
The structures of reference compounds **1**–**12**: Eight flavonoids: Calycosin (**1**), calycosin-7-glucoside (**2**), formononetin (**3**), ononin (**4**), methylnissolin (**5**), astraisoflavan-7-*O*-β-d-glucoside (**6**), 7,2′-dihydroxy-3′,4′-dimethoxy-isoflavane (**7**), and 7,2′-dihydroxy-3′,4′-dimethoxy isoflavan-7-*O*-β-d-glucopyranoside (**8**); and four tri-terpenoid saponins: Astragaloside I (**9**), astragaloside II (**10**), astragaloside III (**11**), and astragaloside IV (**12**).

In this study, an improved UPLC-MS method for simultaneously quantifying twelve major components of AR (including eight flavonoids: Calycosin (**1**), calycosin-7-glucoside (**2**), formononetin (**3**), ononin (**4**), methylnissolin (**5**), astraisoflavan-7-*O*-β-d-glucoside (**6**), 7,2′-dihydroxy-3′,4′-dimethoxy-isoflavane (**7**), and 7,2′-dihydroxy-3′,4′-dimethoxy isoflavan-7-*O*-β-d-glucopyranoside (**8**); and four tri-terpenoid saponins: Astragaloside I (**9**), astragaloside II (**10**), astragaloside III (**11**), and astragaloside IV (**12**)) (Figure 1) was developed, validated, and applied for quantitative evaluation of AR samples sulfur-fumigated with different durations.

## 2. Experimental

### 2.1. Chemicals and Reagents

Twelve reference compounds with purities of >98.0%, including calycosin (**1**), calycosin-7-glu-coside (**2**), formononetin (**3**), ononin (**4**), methylnissolin (**5**), astraisoflavan-7-*O*-β-d-glucoside (**6**), 7,2′-dihydroxy-3′,4′-dimethoxyisoflavane (**7**), 7,2′-dihydroxy-3′,4′-dimethoxy isoflavan-7-*O*-β-d-glu-copyranoside (**8**), astragaloside I (**9**), astragaloside II (**10**), astragaloside III (**11**), and astragaloside IV (**12**), were purchased from the National Institute for the Control of Pharmaceutical and Biological Products (Beijing, China). These compounds showed high stability in methanol solution, of which their structures are shown in Figure 1. Methanol and acetonitrile were of high performance liquid chromatography (HPLC) grade (Tedia Company, Inc., Fairfield, OH, USA). Distilled water was further purified by Milli-Q system (Millipore, Milford, MA, USA); formic acid was purchased from the First Chemical Company of Nanjing (Suzhou, Jiangsu, China); other chemicals were of analytical grade. All solvents and samples were filtered through 0.22 μm membrane filters before injecting into UPLC.

The fresh reference Astragali Radix sample was collected from Inner Mongolia Autonomous region, the indigenous cultivating region of Astragali Radix and authenticated by Prof. Rong-Tao Li. The voucher specimen (AM171114-1) was deposited at the Institute of Medicinal Plant Development, Beijing, China.

### 2.2. Sulfur-Fumigation of AR

The sulfur-fumigated AR samples were self-prepared in our lab from the non-fumigated reference AR sample (AM171114-1) following the modified procedures similar to that by herbal farmers or wholesalers: 50 g AR slices were moistened with 4 mL of water, and left for 0.5 h. Two grams of sulfur powder was heated until burning, then the burning sulfur and the moistened AR slices were carefully put into the lower and upper layer of a desiccator respectively. Seven portions (50 g each portion) were prepared to study of the sulfur-fumigation extent at different collection points of 1, 2, 4, 6, 8, 12, 16, 24, 36, 48, 60, and 72 h, respectively. After fumigation, the AR slices were dried at 40 °C and ground into fine powder.

### 2.3. Instruments

Analyses were carried out using an Agilent 1290 Infinity II RRLC system consisting of a quaternary delivery system, a degasser and an auto-sampler (Agilent Technologies Inc., Santa Clara, CA, USA). Chromatographic separation was achieved on an ACQUITY UPLC^TM^ HSS T3 (100 mm × 2.1 mm, 1.8 μm, Waters, Milford, MA, USA). The column was maintained at 40 °C and eluted at a flowing rate of 0.25 mL/min. The mobile phase consisted of 0.2% formic acid water (A) and acetonitrile (B) using a gradient elution of 1–20% B at 0–5 min, 20–25% B at 5–8 min, 25–30% B at 8–15 min, 30–40% B at 15–18 min, 40–60% B at 18–20 min, 60–90% B at 20–23 min, and washing with 99% B for 23–25 min, equilibration with 1% B at 25–27 min.

An Applied Biosystems 3200 Q-Trap system (AB SCIEX, Framingham, MA, USA) equipped with an electrospray ionization (ESI) source was used and the system was operated in positive and negative mode. Optimization of multiple reaction monitoring (MRM) conditions was carried out with the following source-dependent parameters: Gas 1 and gas 2 were set at 50 psi. The optimized ion spray voltages were set at 5500 V and −4500 V in positive and negative ion mode, respectively. The optimized ion spray voltage and temperature were set at 5500 V and 700 °C, respectively. The operating vaporizer temperature, 500 °C. Nitrogen gas was used in all analyses, and data acquisition and processing were performed using Analyst software version 1.6.2. The MRM parameters are outlined in Table 1.

### 2.4. Standard Solutions Preparation

Standard stock solution 1 and 2 consisted of 7 (**1**–**7**) and 5 (**8**–**12**) accurately weighed reference compounds were directly prepared in methanol, respectively. The final concentrations of these twelve reference compounds in stock solutions were prepared to be 11.88 μg/mL for formononetin, 21.6 μg/mL for calycosin, 17.41 μg/mL for methylnissolin, 15.5 μg/mL for 7,2′-dihydroxy-3′,4′-dimetho-xyisoflavane, 17.44 μg/mL for calycosin-7-glucoside, 38.064 μg/mL for Calycosin-7-glucoside, 6.427 μg/mL for astraisoflavan-7-*O*-β-d-glucoside, 62.4 μg/mL for 7,2′-dihydroxy-3′,4′-dimethoxyisoflavan-7-*O*-β-d-glucopyranoside, 214 μg/mL for astragaloside I, 61.2 μg/mL for astragaloside II, 28.2 μg/mL for astragaloside III and 114.6 μg/mL for astragaloside IV, respectively. The working standard solutions were prepared by diluting the stock solutions with methanol to a series of proper concentrations. The solutions were brought to room temperature and filtered through 0.22 μm membrane filters, and an aliquot of 5 μL was injected into UPLC-MS for subsequent analysis.

### 2.5. Sample Preparation

Methanol extracts: Each AR sample was accurately weighed to approximately 1.0 g and heat refluxed with 50.0 mL of methanol for 4 h. The extract was then filtered using a 0.22 μm PTFE syringe filter before LC-MS analysis.

### 2.6. Method Validation

Method validation assays were carried out according to currently accepted Food and Drug Administration (FDA) guidance. [Guidance for Industry, Bioanalytical Method Validation, US Department of Health and Human Services Food and Drug Administration, Center for Drug Evaluation and Research (CDER), 2001 Center for Veterinary Medicine (CV), 2001, http://www/fda.gov/cder/guidance/index.htm].

#### 2.6.1. Calibration Curves, Limits of Detection and Quantification

The calibration curves for 12 reference compounds were established by plotting peak area ratios of each analyte using the linear regression analysis using 1/X^2^ as a weighting factor. Calibration curves had to have a correlation coefficient (r) of 0.995 or better. The limit of detection (LOD) was determined as signal-to-noise ratio >3 and the limit of quantification (LOQ) was measured as signal-to-noise ratio >10 (Table 2).

#### 2.6.2. Precision, Repeatability and Stability

The intra- and inter-day precision was determined by analyzing 12 analytes from standard stock solution in six replicates during a single day and by duplicating the experiments on three successive days. To further evaluate the repeatability of the developed assays, samples were analyzed in six replicates. Their criteria for acceptability of data were within ±15% relative error (RE) from the nominal values and a precision of within ±15% relative standard deviation (RSD). Stability of AR sample was tested at room temperature and analyzed at 0, 2, 4, 6, 8, 10, 12 and 24 h. The contents of the corresponding compounds were calculated from the corresponding calibration curves.

#### 2.6.3. Recovery Test

The measured recoveries of the compounds were determined by the method of standard addition. Three concentration levels (low, medium, high) of the mixed standard solutions were spiked with a sample of AR, which was analyzed previously using the above described method and the concentration of each component was calculated according to the calibration curves (Table 3).

## 3. Results and Discussion

### 3.1. Optimization of Suitable LC-MS Conditions

**Figure 2:**
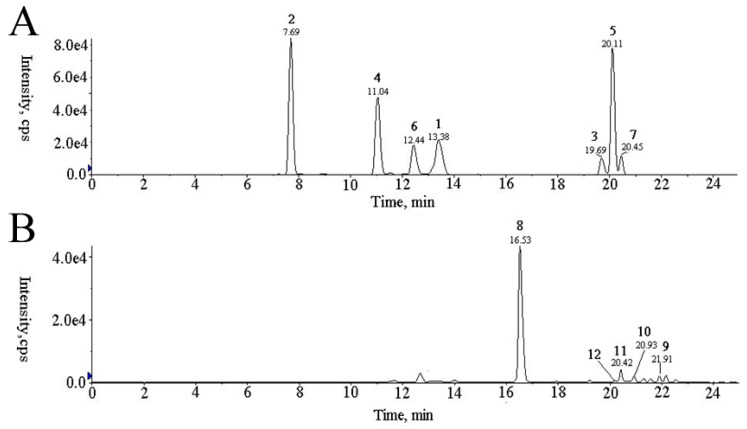
MRM chromatograms in (**A**) positive and (**B**) negative modes. Identification: 1, calycosin; 2, calycosin-7-glucoside; 3, formononetin; 4, ononin; 5, methylnissolin; 6, astraiso-flavan-7-*O*-β-d-glucoside; 7, 7,2′-dihydroxy-3′,4′-dimethoxyisoflavane; 8, 7,2′-dihydroxy-3′,4′-dimethoxy; 8, isoflavan-7-*O*-β-d-glucopyranoside; 9, astragaloside I; 10, astragaloside II; 11, astragaloside III; 12, astragaloside IV.

We initially attempted to optimize one suitable LC-MS method to simultaneously determine all 12 chemical marker compounds in AR. However, we could not obtain acceptable results using one method, where the tested compounds could simultaneously achieve a good ion response in a single ion mode. Thus, two separated batches of analysis were performed under different ion modes. Compounds **1**–**7** and **8**–**12** were performed in positive and negative ion mode, respectively. Figure 2 presented the typical positive base peak intensity (BPI) chromatograms of plasma samples from all the experimental groups.

Meanwhile, two different columns, different mobile phases and detecting ion modes were tested during method development. The selection of UPLC columns with high separation efficiency is a prerequisite. Here, two chromatographic columns, ethylene bridged hybrid (BEH) C18 column (2.1 mm × 100 mm, 1.7 μm, Waters) and high strength silica (HSS) T3 column (2.1 mm × 100 mm, 1.8 μm, Waters), were utilized to investigate for the comprehensive metabolome. The BEH C18 column is one universal column choice for UHPLC separations. While HSS T3 column with 100% silica particle, is used to retain and separate smaller, more water-soluble polar organic compounds than the BEH C18 column (Zhao et al., 2013). The result showed that HSS T3 column could gain a better chromatographic separation for the 12 tested analysts. 

Mobile phases including acetonitrile-water and methanol-water with modifiers such as acetic acid, formic acid, and different gradient elution modes were all investigated. The results showed that a mobile phase consisting of water (0.2% formic acid) and acetonitrile (0.2% formic acid) gave the best separation.

### 3.2. Method Validation

#### 3.2.1. Calibration Curves, Limits of Detection and Quantification

Standard stock solutions were prepared as described and diluted to appropriate concentrations to establish the calibration curves. At least six different concentrations were analyzed in triplicate, and the calibration curves were then constructed by plotting the peak areas vs. the concentration of each analyte. As shown in Table 2, all the analytes showed good linearity (R^2^ ≥ 0.9986) in a relatively wide concentration range. The analysis of LOD and LOQ also showed a well quantification, which ranged from 0.001–1.55 ng/mL and 0.01–3.10 ng/mL, respectively.

#### 3.2.2. Precision, Repeatability and Stability

The precisions were determined by analyzing known concentrations of the 12 analytes from two standard stock solutions in six replicates during a single day and by duplicating the experiments. To further evaluate the repeatability of the developed assays, samples were analyzed in six replicates as described above. Stability of AR samples was tested at room temperature and analyzed at different time points within one day. The contents of the 12 analytes were calculated from the corresponding calibration curves. Table 2 indicated that the RSD values for measurement precision, repeatability and stability of the 12 compounds were all less than 5.0%, which demonstrates good precision, repeatability and stability of the developed method.

#### 3.2.3. Accuracy

Accuracy of the analytical method was evaluated by measuring percentage recovery of 12 analytes. The results of the recovery test are shown in Table 3, which all ranged from 95–105% at three spiked concentrations.

### 3.3. Quantification of the Major Components in AR with and without Sulfur-Fumigation

The validated LC-MS method was applied for quantitative determination of the 12 components with and without sulfur-fumigation. The contents of the eight flavonoids and four triterpenoid saponins were summarized in Table 4. From the results, it can be found that compared with the non-fumigated sample, the contents of two flavonoids calycosin (**1**) and formononetin (**3**) decreased significantly ranging from 39.2% to 45.4% and 35.5% to 40.5%, respectively; 7,2′-dihydroxy-3′,4′-dimethoxyisoflavane (**7**) had a large fluctuation ranging from 6.5% to 39.8%; the content of methylnissolin (**5**) had no obvious change in the sulfur-fumigated samples; while the contents of four flavonoid glycosides (compounds **2**, **4**, **6**, and **8**) all increased remarkably which suggests the occurence of chemical transformation of flavonoids and glycosides in the sulfur-fumigated samples. In addition, the contents of astragaloside III (**11**) and astragaloside IV (**12**) decreased moderately ranging from 11.5% to 40.0% and 15.5% to 47.7%, respectively, when compared with the non-fumigated sample; the content of astragaloside I (**9**) displayed no obvious change in the sulfur-fumigated sample; and the content of astragaloside II (**10**) was not detected because of the limited detection. Furthermore, the analyses of the detected compounds’ contents over different sulfur-fumigated times suggested that the reduction proportions of compounds **7**, **11**, and **12** had a proportional relationship with sulfur-fumigated time. All above results indicated that sulfur-fumigation can decrease the contents of partial aglycones and triterpenoid saponins and increase the contents of flavonoid glycosides in AR significantly. Therefore, it could be concluded that sulfur-fumigation can significantly influence the inherent quality of the raw materials of AR.

### 3.4. General Procedure for the Synthesis of Flavonoid Glycosides

The variation of flavonoid and glycoside content after the sulfur-fumigation of AR compared with the reference sample suggest that the flavonoids may have a reaction with glucoses under the high temperature and acidic conditions that occur during the sulfur-fumigation process. In order to confirm the deduction, we further designed a procedure for the synthesis of flavonoid glycosides which was similar to the sulfur-fumigation circumstances.

**Figure 3:**
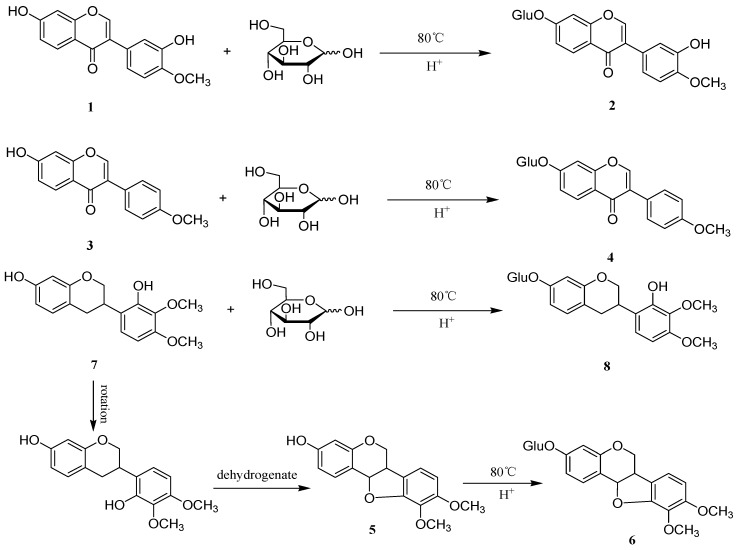
The synthesis of flavonoid glycosides.

Calycosin (**1**), formononetin (**3**), and 7,2′-dihydroxy-3′,4′-dimethoxyisoflavane (**7**) (10 mg each) were dissolved with mixed solvent DMSO and H_2_O (2 mL each) in the sealing tubes, respectively. Then 1 mL of concentrated hydrochloric acid and D-glucose (10 mg, 0.055 mmol) were added. The reaction mixture was heated to 80 °C. After 12 h, the four reaction mixtures were analyzed by HPLC, respectively. The results showed that calycosin-7-glucoside (**2**), ononin (**4**), astraisoflavan-7-*O*-β-d-glucoside (**6**), and 7,2′-dihydroxy-3′,4′-dimethoxy isoflavan-7-*O*-β-d-glucopyranoside (**8**) were generated by comparison with the standard materials (Figure 3). This experiment further confirmed that sulfur-fumigation can increase the extent of transformation of flavonoids to flavonoid glycosides.

## 4. Conclusions

In the present study, a LC-MS method was established for simultaneous quantification of twelve major components in AR, and successfully applied for quantitatively evaluating the effects of sulfur-fumigation on the quality of AR. Compared with previously reported methods, the newly developed method used MRM mode of LC-MS which was the first application to simultaneously detect flavonoids and triterpenoid saponins in *A. mongholicus*.

In this study, it was observed that the content of the major flavonoids decreased significantly, while the corresponding glycosides increased accordingly when compared with non-fumigated AR. The contents of the major triterpene glycosides also decreased in the sulfur-fumigation samples, but the degree of reductions were limited. Sulfur-fumigation can influence not only the content of the components in AR, but also the chemical transformation of flavonoids and glycosides. It was suggested that sulfur-fumigation should be forbidden for processing and conservation of Chinese medicinal herbs before the efficacy and safety of sulfur-fumigated herbs are systematically investigated. Alternatives to sulfur-fumigation for processing and conservation of AR should also be further developed.

## Figures and Tables

**Table 1:** Multiple reaction monitoring (MRM) transitions and parameters for the detection of the 12 analytes.

No.	Analyte	Precursor Ion (*m*/*z*)	Product Ion (*m*/*z*)	DP ^a^ (V)	CE ^b^ (V)
1	Calycosin	285.2	213.1	70	45
2	Calycosin-7-glucoside	447.3	285.4	60	20
3	Formononetin	269.0	167.2	70	47
4	Ononin	431.1	269.1	65	20
5	Methylnissolin	301.2	167.2	54	20
6	Astraisoflavan-7-*O*-β-d-glucoside	463.3	167.4	60	40
7	7,2′-dihydroxy-3′,4′-dimethoxyisoflavane	303.0	167.2	55	19
8	7,2′-dihydroxy-3′,4′-dimethoxy isoflavan-7-*O*-β-d-glucopyranoside	463.3	301.0	−74	−24
9	Astragaloside I	867.9	59.1	−250	−83
10	Astragaloside II	825.7	59.1	−110	−70
11	Astragaloside III	783.8	160.9	−150	−47
12	Astragaloside IV	783.8	101.0	−115	−57

^a^ declustering potential; ^b^ collision Energy.

**Table 2:** Validation with respect to linearity, limit of quantification (LOQ), limit of detection (LOD), precision, repeatability and stability.

Analytes	Regression Equation (μg/mL)	Linear Range (μg/mL)	Correlation Coefficient (R2)	LOQ (ng/mL)	LOD (ng/mL)	Precision RSD ^a^ (%)		Repeatability RSD (%)	Stability RSD (%)
						Intra-Day	Inter-Day		
Calycosin	y = 446765x + 322603	0.4752~11.88	0.9989	0.59	0.06	2.06%	3.16%	4.47%	3.93%
Calycosin-7-glucoside	y = 202558x + 365088	0.864~21.6	0.9992	1.08	0.11	1.31%	2.09%	3.43%	2.95%
Formononetin	y = 469388x + 141426	0.3482~17.41	0.9986	0.87	0.35	1.65%	1.69%	4.09%	3.37%
Ononin	y = 51545x + 4316.5	0.31~15.5	0.9994	3.10	1.55	1.42%	3.86%	4.63%	4.05%
Methylnissolin	y = 329224x + 84783	0.6976~17.44	0.9993	0.09	0.02	1.46%	2.53%	4.31%	2.99%
Astraisoflavan-7-*O*-β-d-glucoside	y = 130675x + 208291	1.5226~38.064	0.9991	0.08	0.04	2.03%	2.78%	4.49%	3.73%
7,2′-dihydroxy-3′,4′-dimethoxyisoflavane	y = 57437x + 11250	0.2571~6.427	0.9990	0.32	0.35	1.96%	3.53%	4.92%	3.77%
7,2′-dihydroxy-3′,4′-dimethoxy isoflavan-7-*O*-β-d-glucopyranoside	y = 127060x + 236161	2.496~62.4	0.9988	0.01	0.001	1.25%	2.37%	3.68%	1.50%
Astragaloside I	y = 1009.3x − 10607	8.56~214	0.9991	0.09	0.04	2.36%	3.18%	4.84%	2.52%
Astragaloside II	y = 10750x + 25435	1.028~25.2	0.9993	0.03	0.002	1.65%	3.78%	2.66%	2.03%
Astragaloside III	y = 12640x + 30515	1.128~28.2	0.9993	0.03	0.01	1.41%	2.19%	2.65%	1.87%
Astragaloside IV	y = 6748.6x + 164720	4.584~114.6	0.9989	0.06	0.01	2.18%	1.98%	4.67%	2.06%

^a^ relative standard deviation.

**Table 3:** Results of recovery.

Analyte	Initial Amount (μg)	Added Amount (μg)	Detected Amount (μg)	Recovery (%)	RSD (%)
		0.61	1.90	103.50%	3.2
Calycosin	1.22	1.22	2.48	101.50%	1.5
		1.83	3.01	98.50%	2.3
		3.735	4.79	96.70%	2.7
Calycosin-7-glucoside	7.47	7.47	14.51	97.10%	0.9
		11.205	19.28	103.20%	1.7
		1.21	3.47	95.40%	1.6
Formononetin	2.42	2.42	5.10	105.20%	3.1
		3.63	6.01	99.20%	2.7
		0.87	2.61	99.60%	0.4
Ononin	1.74	1.74	3.53	101.40%	0.9
		2.61	4.56	104.70%	1.5
		2.29	7.12	103.90%	3.6
Methylnissolin	4.57	4.57	9.36	102.30%	3.7
		6.86	12.06	105.50%	3.0
		8.54	26.67	104.10%	2.6
Astraisoflavan-7-*O*-β-d-glucoside	17.08	17.08	34.02	99.60%	3.4
		25.62	42.23	98.90%	2.6
		1.99	6.24	104.50%	1.5
7,2′-dihydroxy-3′,4′-dimethoxyisoflavane	3.98	3.98	8.15	102.30%	1.6
		5.97	10.11	101.60%	3.2
		1.26	3.87	102.70%	3.1
7,2′-dihydroxy-3′,4′-dimethoxy	2.51	2.51	4.96	98.80%	2.3
		3.77	6.39	101.80%	5.7
isoflavan-7-*O*-β-d-glucopyranoside		2.01	6.12	101.70%	0.6
Isoflavan-7-*O*-β-d-glucopyranoside	4.01	4.01	7.90	98.40%	2.3
		6.02	9.58	95.50%	2.6
		1.23	3.79	103.10%	3.1
Astragaloside I	2.45	2.45	4.88	99.60%	4.1
		3.68	6.06	98.90%	2.8
		1.07	3.16	98.60%	1.5
Astragaloside III	2.14	2.14	4.40	102.90%	2.7
		3.21	5.30	99.10%	3.1
		4.33	13.50	104.10%	1.8
Astragaloside IV	8.65	8.65	16.83	97.30%	3.1
		12.98	22.44	103.80%	2.2

**Table 4:** The contents of twelve reference compounds in AR with and without sulfur-fumigation (mg/g, *n* = 3).

Compounds	AR with and without Sulfur-Fumigation.												
	1 h	2 h	4 h	6 h	8 h	12 h	16 h	24 h	36 h	48 h	60 h	72 h	Non-Fumigated
**1**	7.49 ± 0.03	5.04 ± 0.04	6.40 ± 0.02	7.36 ± 0.07	4.91 ± 0.00	5.97 ± 0.01	7.44 ± 0.10	5.37 ± 0.02	5.42 ± 0.05	4.86 ± 0.01	5.28 ± 0.06	6.03 ± 0.03	13.55 ± 0.68
**2**	4.24 ± 0.05	5.45 ± 0.02	5.10 ± 0.00	4.83 ± 0.01	4.61 ± 0.04	4.27 ± 0.02	4.07 ± 0.05	4.39 ± 0.03	4.33 ± 0.04	4.48 ± 0.01	3.92 ± 0.02	3.87 ± 0.02	3.21 ± 0.01
**3**	0.59 ± 0.01	0.59 ± 0.03	0.75 ± 0.02	0.78 ± 0.00	0.50 ± 0.02	0.64 ± 0.01	0.50 ± 0.03	0.62 ± 0.00	0.60 ± 0.02	0.56 ± 0.01	0.61 ± 0.02	0.63 ± 0.01	1.37 ± 0.04
**4**	0.81 ± 0.02	1.19 ± 0.09	1.04 ± 0.01	1.05 ± 0.04	0.88 ± 0.05	0.80 ± 0.07	1.06 ± 0.02	0.89 ± 0.00	0.91 ± 0.02	0.95 ± 0.01	0.76 ± 0.02	0.75 ± 0.01	0.40 ± 0.02
**5**	0.30 ± 0.01	0.25 ± 0.00	0.27 ± 0.02	0.27 ± 0.00	0.21 ± 0.00	0.22 ± 0.01	0.35 ± 0.02	0.22 ± 0.05	0.23 ± 0.01	0.22 ± 0.02	0.23 ± 0.00	0.22 ± 0.01	0.24 ± 0.01
**6**	0.55 ± 0.00	0.90 ± 0.01	0.69 ± 0.01	0.64 ± 0.02	0.59 ± 0.00	0.58 ± 0.05	0.53 ± 0.07	0.58 ± 0.00	0.52 ± 0.02	0.67 ± 0.00	0.47 ± 0.00	0.44 ± 0.01	0.32 ± 0.02
**7**	4.18 ± 0.06	2.61 ± 0.01	2.99 ± 0.05	3.44 ± 0.00	2.93 ± 0.02	3.29 ± 0.04	3.06 ± 0.01	2.51 ± 0.00	3.17 ± 0.02	2.60 ± 0.00	2.54 ± 0.00	3.24 ± 0.01	4.47 ± 0.10
**8**	0.86 ± 0.00	1.59 ± 0.00	1.38 ± 0.05	1.20 ± 0.07	1.13 ± 0.06	1.16 ± 0.02	0.95 ± 0.01	1.27 ± 0.00	1.08 ± 0.04	1.42 ± 0.06	0.97 ± 0.00	0.75 ± 0.00	0.68 ± 0.00
**9**	0.71 ± 0.01	0.74 ± 0.02	0.70 ± 0.00	0.71 ± 0.05	0.70 ± 0.00	0.70 ± 0.02	0.74 ± 0.04	0.69 ± 0.07	0.70 ± 0.00	0.68 ± 0.01	0.67 ± 0.02	0.70 ± 0.04	0.73 ± 0.04
**10**	ND	ND	ND	ND	ND	ND	ND	ND	ND	ND	ND	ND	ND
**11**	0.78 ± 0.06	0.84 ± 0.02	0.79 ± 0.01	0.70 ± 0.04	0.71 ± 0.00	0.73 ± 0.01	0.75 ± 0.00	0.59 ± 0.02	0.60 ± 0.00	0.64 ± 0.01	0.57 ± 0.02	0.58 ± 0.01	0.95 ± 0.03
**12**	3.97 ± 0.00	4.17 ± 0.02	3.73 ± 0.01	3.38 ± 0.01	3.59 ± 0.00	3.53 ± 0.02	3.61 ± 0.01	2.68 ± 0.05	2.81 ± 0.02	3.03 ± 0.04	2.62 ± 0.00	2.58 ± 0.01	4.94 ± 0.02
